# Inflammatory Cytokines That Enhance Antigen Responsiveness of Naïve CD8^+^ T Lymphocytes Modulate Chromatin Accessibility of Genes Impacted by Antigen Stimulation

**DOI:** 10.3390/ijms232214122

**Published:** 2022-11-16

**Authors:** Akouavi Julite Irmine Quenum, Madanraj Appiya Santharam, Sheela Ramanathan, Subburaj Ilangumaran

**Affiliations:** Department of Immunology and Cell Biology, Faculty of Medicine and Health Sciences, Université de Sherbrooke, Sherbrooke, QC J1H 5N4, Canada

**Keywords:** inflammatory cytokines, IL-15, IL-21, naïve CD8^+^ T cells, TCR, cytokine priming, ATACseq, gene expression

## Abstract

Naïve CD8^+^ T lymphocytes exposed to certain inflammatory cytokines undergo proliferation and display increased sensitivity to antigens. Such ‘cytokine priming’ can promote the activation of potentially autoreactive and antitumor CD8^+^ T cells by weak tissue antigens and tumor antigens. To elucidate the molecular mechanisms of cytokine priming, naïve PMEL-1 TCR transgenic CD8^+^ T lymphocytes were stimulated with IL-15 and IL-21, and chromatin accessibility was assessed using the assay for transposase-accessible chromatin (ATAC) sequencing. PMEL-1 cells stimulated by the cognate antigenic peptide mgp100_25-33_ served as controls. Cytokine-primed cells showed a limited number of opening and closing chromatin accessibility peaks compared to antigen-stimulated cells. However, the ATACseq peaks in cytokine-primed cells substantially overlapped with those of antigen-stimulated cells and mapped to several genes implicated in T cell signaling, activation, effector differentiation, negative regulation and exhaustion. Nonetheless, the expression of most of these genes was remarkably different between cytokine-primed and antigen-stimulated cells. In addition, cytokine priming impacted the expression of several genes following antigen stimulation in a synergistic or antagonistic manner. Our findings indicate that chromatin accessibility changes in cytokine-primed naïve CD8^+^ T cells not only underlie their increased antigen responsiveness but may also enhance their functional fitness by reducing exhaustion without compromising regulatory controls.

## 1. Introduction

CD8^+^ T lymphocytes confer immune protection against viral and bacterial pathogens, as well as tumor development and progression [1,2]. To carry out these functions, naïve CD8^+^ T cells must receive two essential signals delivered via the T cell antigen receptor (TCR) (signal 1) and costimulatory receptors such as CD28 (signal 2) [3]. Innate immune responses often precede antigen (Ag)-specific adaptive immune responses mediated by T cells [4]. The activation of innate immune cells via pattern recognition receptors generates inflammatory cytokines, chemokines and other effector molecules [5,6,7]. In addition to recruiting T cells and upregulating costimulatory ligands on antigen presenting cells (APC), the inflammatory mediators of the innate immune response may directly contribute to T cell activation and boost their effector functions. The molecular events that accompany the transition from innate to adaptive immune response continue to be an important area of investigation due to their relevance to vaccinology, autoimmunity and antitumor immunity [8,9,10].

Inflammatory cytokines such as type-I interferons (IFN-I), interleukin-12 (IL-12), IL-27 and IL-21 have been shown to provide a ‘third signal’ to activated CD8^+^ T cells, stimulating more efficient clonal expansion and effector functions [11,12,13,14,15,16,17]. Distinct from the third signal, we and others have shown that certain cytokines produced during innate immune responses can act in synergistic combinations to stimulate Ag non-specific proliferation of naïve CD8^+^ T cells independently of TCR and costimulatory signaling [18,19,20]. These stimulatory cytokines include inflammatory cytokines such as IL-6 and IL-21, and cytokines that sustain T cell homeostasis such as IL-7 or IL-15, which are also induced by innate immune stimulation [21,22]. Importantly, naïve CD8^+^ T cells pre-stimulated with IL-21 and IL-15 display increased sensitivity to Ag, proliferate strongly and exhibit potent Ag-specific cytolytic activity upon subsequent Ag stimulation [19]. Moreover, cytokine-primed CD8^+^ T cells gain sensitivity toward altered peptide ligands of lower affinity than the cognate peptide [23]. Indirect evidence suggests that such an inflammatory cytokine driven increase in the Ag responsiveness of CD8^+^ T cells also occurs in vivo [24,25]. Therefore, the ability of the inflammatory cytokines to boost Ag-specific responses of naïve CD8^+^ T cells, referred to here as ‘cytokine priming’, can play a direct role in shaping the adaptive immune response mediated by CD8^+^ T cells, which may have implications for immunity, autoimmunity and antitumor immunity [26].

The molecular mechanisms underlying the heightened antigen responsiveness of cytokine primed naïve CD8^+^ T cells remains unclear. We have shown that cytokine priming is accompanied by profound changes in the expression of several cell surface molecules implicated in T cell activation such as CD5, CD8, CD44, CD132, CD134 (OX40) and GITR (TNFRSF18) [19,27]. However, all these changes were also induced by homeostatic cytokines alone even in the absence of synergistic stimulation by inflammatory cytokines. In addition, the cytokine induced augmentation of TCR signaling can occur even without costimulation [19,27]. We have shown that inflammatory cytokines enhanced the activation of STAT5 induced by homeostatic cytokines and its DNA-binding activity [19]. Moreover, virus-induced inflammation was shown to increase the activation of proximal TCR signaling molecules ZAP70 and PLCγ, and ERK following TCR stimulation [25].

The above studies suggest that cytokine priming by inflammatory and homeostatic cytokines induce a ‘poised state’ in naïve CD8^+^ T cells that allows for them to robustly respond upon subsequent encounter with antigen. To gain molecular insight into this poised state, we compared the chromatin accessibility of naïve, cytokine-primed (CytP) and Ag-stimulated (AgS) CD8^+^ T cells.

## 2. Results

### 2.1. Comparison of ATACseq Peaks Modulated by Cytokine Priming and Ag Stimulation

**Figure 1:**
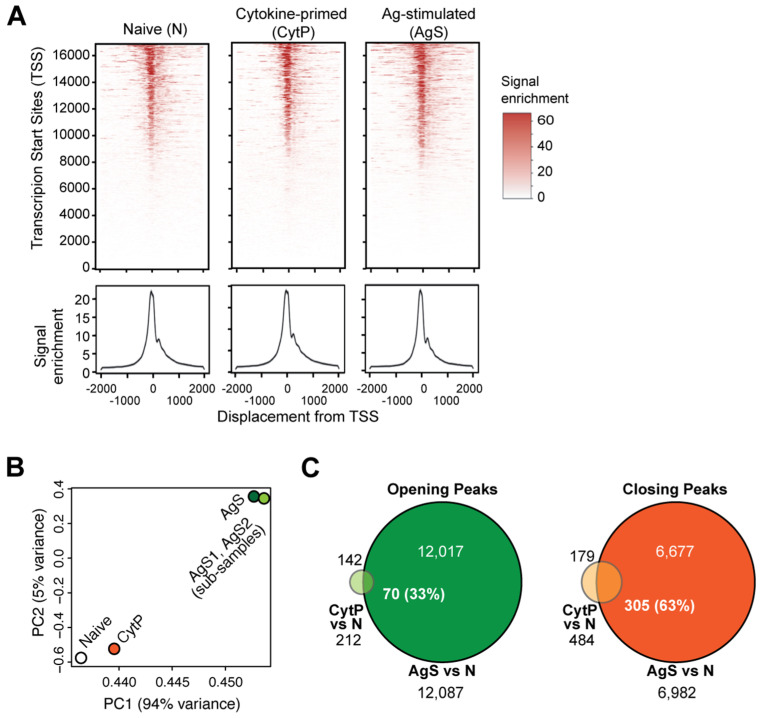
Cytokine-priming of CD8^+^ T cells modulates a limited number of ATACseq peaks compared to Ag-primed cells but show substantial overlap with the latter. (**A**) Fragment length distribution analysis of naïve (N), cytokine-primed (CytS) and Ag-stimulated (AgS) PMEL-1 TCR transgenic CD8^+^ T cells. (**B**) Principal component analysis of the ATACseq reads of N, CytP and AgS cells. AgS1 and AgS2 represent random subgrouping of reads from AgS cells to ensure the random distribution of opening and closing ATACseq peaks (also see Supplementary Figure S1). (**C**) Overlap between the opening and closing peaks of CytP and AgS cells compared to naïve cells.

A role for STAT5 in promoting T helper cell differentiation via the modulation of chromatin accessibility has also been documented [28,29,30]. These reports raised the possibility that cytokine priming may induce changes in chromatin accessibility that would facilitate and strengthen TCR signaling, and thereby increase the Ag sensitivity of CytP naïve CD8^+^ T cells. To test this hypothesis, we evaluated genome-wide transcription factor (TF) occupancy in PMEL-1 TCR transgenic CD8^+^ T cells stimulated by IL-15 and IL-21, or with the cognate antigenic peptide by the assay for transposase-accessible chromatin sequencing (ATACseq). Fragment length distribution analysis showed the enrichment of ATACseq reads around TSS spanning approximately 200 bp covering a mono-nucleosomal peak in all three samples (Figure 1A). Principal component (PC) analysis of ATACseq reads revealed that CytP cells were more closely related to naïve T cells than AgS cells (Figure 1B). AgS cells showed 12,087 opening and 6982 closing peaks with a rlog fold change value of 1.5 compared to naïve cells, whereas the CytP cells showed only 212 opening peaks and 484 closing peaks (Figure 1C). Nevertheless, 70 of the opening peaks (33%) and 305 of the closing peaks (63%) in CytP cells were represented in AgS cells (Figure 1C), suggesting that cytokine priming alters the accessibility of a subset of genes that are modulated by TCR signaling.

### 2.2. Comparison of ATACseq Peaks Modulated by Cytokine Priming and Ag Stimulation

**Figure 2:**
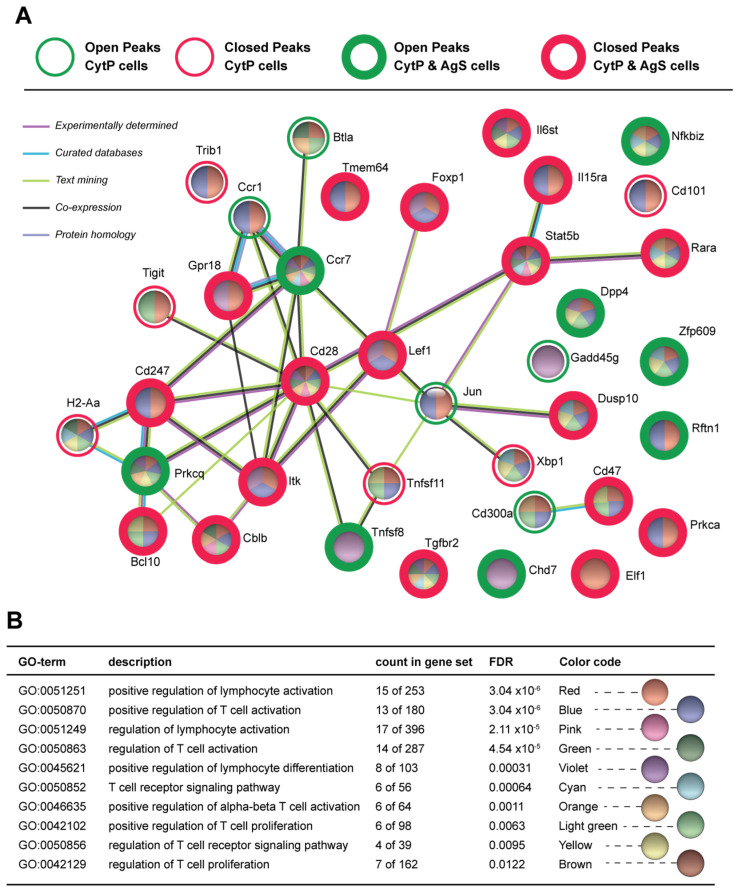
Chromatin accessibility of genes implicated in T cell activation, effector functions and regulation in cytokine-primed and Ag-stimulated cells. (**A**) Protein network analysis of genes within the GO groups related to T lymphocyte activation near the opening or closing peaks in CytP and AgS cells using the STRING database. Open and closed peaks are indicated by green and red colored circles, respectively. Peaks modulated in CytP cells alone are indicated by thin circles, and those modulated in both CytP and AgS cells by thick circles. The pie diagrams for individual genes are again color-coded based on their inclusion within the various gene ontology (GO) groups listed in (**B**). Only the GO groups that show a significantly low false discovery rate (FDR) are shown.

The opening and closing chromatin accessibility peaks found in CytP and AgS cells were annotated using HOMER to associate the peaks with nearby genes. The open peaks found in CytP cells, AgS cells and both cells are listed in Supplementary Table S1A–C, and the closing peaks in these three groups of cells are given in Supplementary Table S2A–C. Proteins encoded by genes nearest to the opening and closing peaks of CytP cells were analyzed using the STRING database to study their functional enrichment based on biological processes. This analysis identified several Gene Ontology (GO) groups related to T cell activation, differentiation and functions that are modulated in CytP cells (Table 1A and Table S3). Similar observations were made of opening and closing peaks found in both CytP cells and in AgS cells (Table 1B and Table S4). Protein network analysis of all genes near the opening or closing peaks in CytP cells revealed a complex network (Supplementary Figure S2). Therefore, we restricted this analysis to genes within the GO groups related to T lymphocyte activation to understand how cytokine priming could influence TCR signaling (Figure 2A). When analyzed within the opening and closing peaks of both CytP and AgS cells, these GO enrichment groups showed a significantly low false discovery rate (FDR; Figure 2B). Among the genes that were modulated in both CytP and AgS cells, the opening peaks were found adjacent to *Rftn1* (Raftlin 1), *Dpp4* (Dipeptidylpeptidase 4; CD26), *Pkrcq* (PKCq), *Chd7*, *Ccr7*, *Nfkbiz* (IκBζ), *Tnfsf8* (CD153) and *Zfp609*, whereas closing peaks were found near several other genes (*Cd247, Itk, Bcl10, Elf1, Cd28, Dusp10, Foxp1, Gpr18, Il15ra, Il6st, Lef1, Prkca, Rara, Stat5b, Tgfbr2, Cblb, CD47* and *Tmem64*) (Figure 2A, Table 2). The functions of these genes are related to T lymphocyte activation, signaling, effector differentiation, negative regulation and exhaustion, as detailed in Table 2. Cytokine priming also modulated chromatin accessibility near a few other genes that fall within the GO terms of T lymphocyte functions but were not significantly affected in AgS cells (*Btla, Gadd45b, Cd300a, Jun, Xbp, Tnfsf11, Cd101*) (Figure 2A and Table 2).

### 2.3. Chromatin Accessibility in Ag-Stimulated Cells

**Figure 3:**
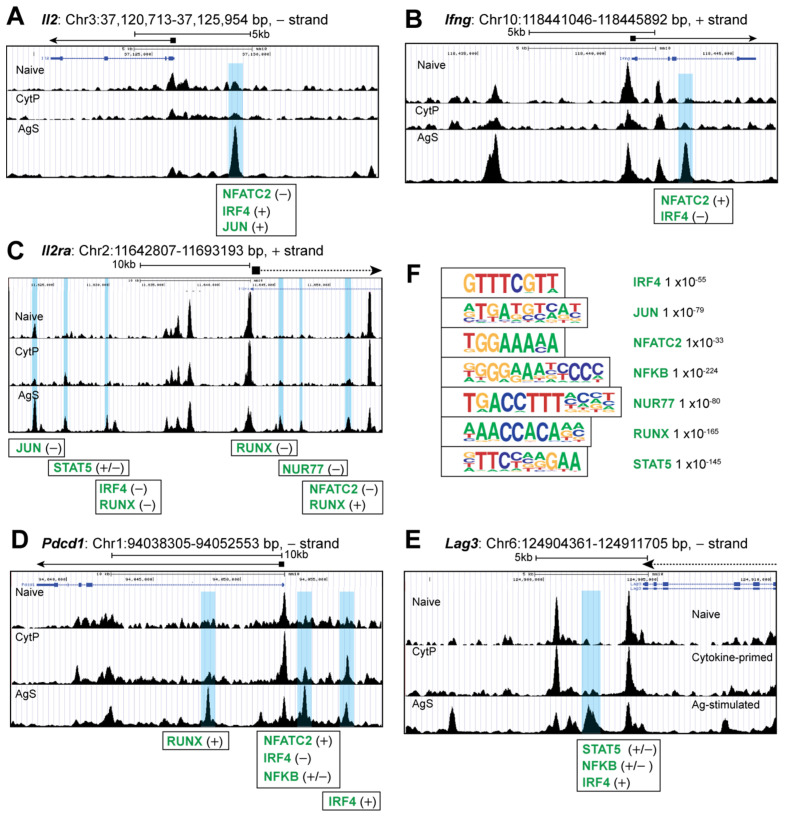
Chromatin accessibility in Ag-stimulated cells. Genome browser snapshots of chromatin accessibility signal at (**A**) *Il2*, (**B**) *Ifng* and (**C**) *Il2ra*, (**D**) *Pdcd1* and (**E**) *Lag3* genes. The chromosomal locations of the genes, accessibility peaks opening only in AgS cells (shaded blue) and the corresponding transcription factor bindings sites are indicated. Position of genes are indicated by solid lines for full genes and dotted lines for partially covered genes within the genome area shown. The peak heights (FC signal) are adjusted to the same value for all three tracks before taking the snapshots. (**F**) The transcription factors binding motifs that are significantly enriched in chromosome accessibility peaks opening in AgS cells.

To understand how cytokine priming modulates chromatin accessibility, we first analyzed the ATACseq peaks near the gene loci that are known to be modulated by Ag stimulation in CD8^+^ T cells. As expected, a strong modulation of chromatin accessibility was observed near several genes such as *Il2, Ifng* and *Il2ra*, as indicated by the opening peaks that correspond to the binding sites of several TF activated by TCR signaling including NFATc, IRF4, Jun, Nur77 and RUNX (Figure 3A–D). Genes coding for the checkpoint inhibitors PD1 (*Pdcd1*) and LAG3, which are strongly induced following TCR stimulation, also showed marked changes in chromatic accessibility with opening peaks for NFATc, NF-κB, IRF4, STAT5 and RUNX (Figure 3E,F). Notably, almost none of these sites were accessible in CytP cells, indicating that cytokine priming does not induce many of the genes that promote effector cell differentiation. AgS cells also showed several other opening peaks (*Cd3e*), closing peaks (*Il7r*, *P2rx7*) or both (*Ctla4*), that were not affected in CytP cells (Supplementary Figure S3).

### 2.4. Chromatin Accessibility in Cytokine-Primed Cells

**Figure 4:**
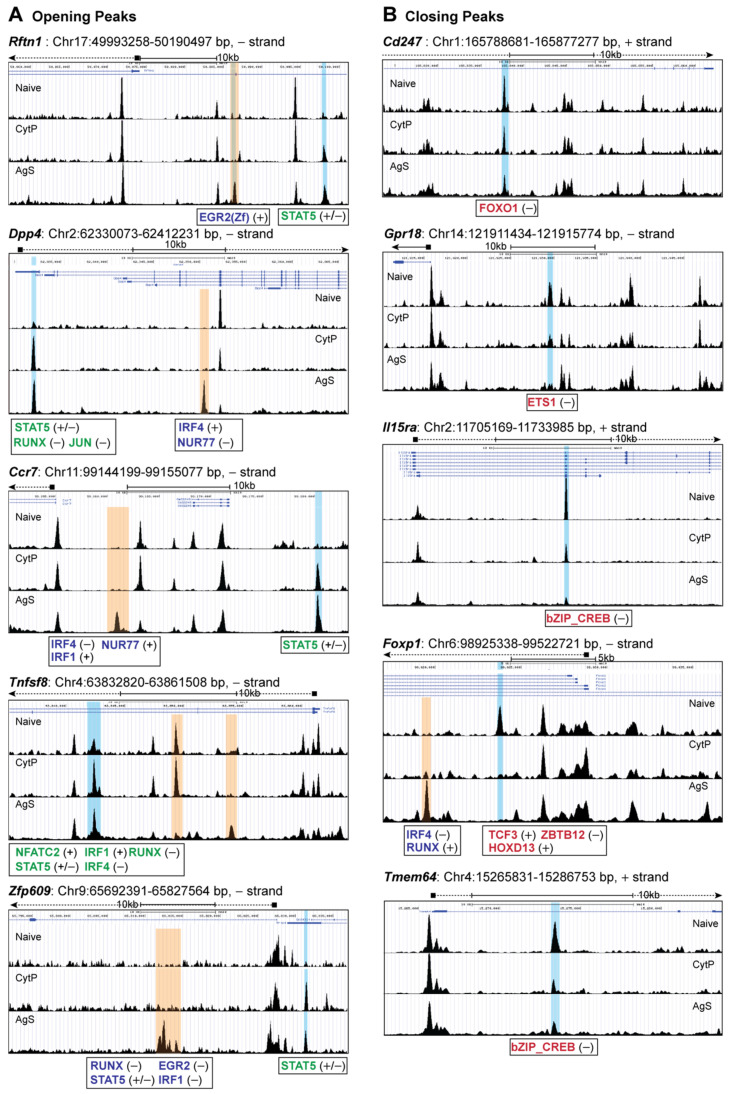
Chromatin accessibility in cytokine-primed cells. Genome browser snapshots of chromatin accessibility signal found in the opening (**A**) and closing (**B**) peaks of CytP cells that also occurred in AgS cells. Peaks modulated in both CytP and Ags cells are shaded blue, whereas those modulated only in AgS cells are shaded yellow. Color schemes for transcription factor binding motifs: green, in the opening peaks of CytP and AgS cells; red, in the closing peaks of CytP and AgS cells; blue, in the opening peaks of only AgS cells.

We next examined the chromatin accessibility sites in CytP cells near genes implicated in T lymphocyte activation and functions, many of which are also modulated in a similar fashion in AgS cells (Table 2). These sites included many closing peaks and fewer opening peaks. An analysis of chromatin accessibility near these genes revealed similar changes of comparable magnitude in the opening peaks near *Rftn1* (immune synapse component), *Dpp4* (modulator of TCR signaling), *Ccr7* (cytoskeletal remodeling and cell migration), *Tnfsf8* (T cell proliferation) and *Zfp609* (repressor of *Rag* genes) genes (Table 2, Figure 4A). Whereas all the above opening peaks harbored the STAT5b binding motif, some of these opening peaks (Eg., *Dpp4, Tnfsf8*) also contained motifs for other TF such as NFAT, RUNX and IRF4 that are also found near the opening peaks of AgS cells. Strikingly, the majority of these genes also showed additional opening peaks only in AgS cells that contained motifs for various TF (Figure 4A), suggesting that these genes are likely expressed in Ag-stimulated cells, whereas they are poised for expression in CytP cells.

Analysis of the peaks closing in both CytP and AgS cells (Table 2, Figure 4B) revealed similar changes near *Cd247* (CD3ζ, the signal transducing chain of the TCR), *Gpr18*, *Il15ra* (controls memory CD8 T cell generation and homeostasis), *Foxp1* (maintains quiescence in naïve T cells), *Tmem64* (unknown function in T cells; possibly involved in calcium signaling) genes. These peaks contained motifs for various TF such as FOXO1, ETS1, bZIP-CREB, TCF3 and HOXD13. Notably, the binding motifs for bZIP-CREB and ETS1 are found in several other closing peaks of both CytP and in AgS cells or only in the latter (Supplementary Figure S4). However, unlike for opening peaks, additional changes in chromatin accessibility near these closing peaks were uncommon.

### 2.5. Transcription Factor Binding Motifs Enriched in CytP and AgS Cells

**Figure 5:**
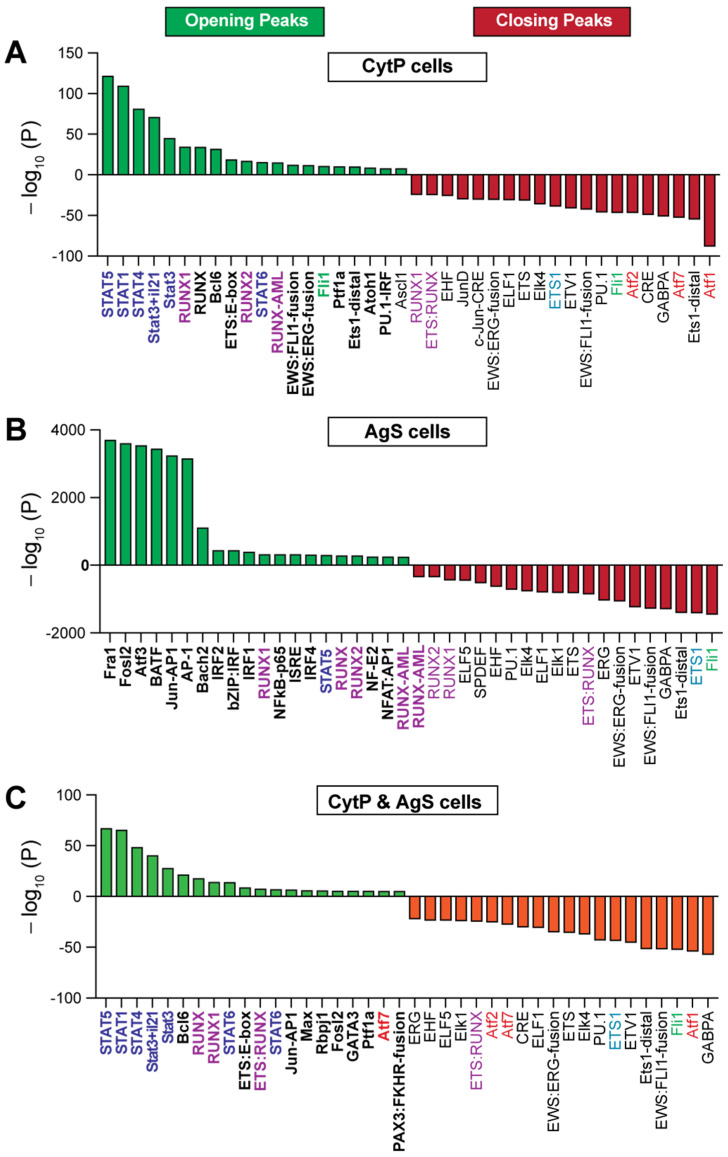
Enrichment of transcription factor binding motifs in cytokine-primed and Ag-stimulated cells. Top twenty transcription factor binding motifs found near the opening and closing peaks of CytP (**A**) and AgS (**B**) cells. Motifs found near the opening and closing peaks of both CytP and AgS cells are shown in (**C**).

Consistent with stimulation by IL-15 and IL-21, CytP cells showed an enrichment of binding sites for the STAT proteins STAT5, STAT1, STAT3 at the opening peaks (Figure 5A; Supplementary Table S5A), although STAT4 and STAT6 binding was also observed. On the other hand, AgS cells predominantly showed motifs for the beta leucine zipper (bZIP) containing TF FRA1 (FOSL1), FOSL2, ATF3, BATF and AP1 (Figure 5B; Supplementary Table S5B). Several other TF motifs such as the ones for Tbet and Eomes, which are known to be activated by Ag, are also significantly enriched in AgS cells (Supplementary Table S5B). The closing peaks of CytP and AgS cells remarkably differed in their accessibility to TF binding motifs, with the activating TF (ATF) family members ATF1, ATF7 and ATF2 dominating in CytP cells in contrast to the ETS (E26 transformation-specific or E-twenty-six) family members FLI1 and ETS1 dominating in AgS cells (Figure 5A,B; Supplementary Table S6A,B). However, analysis of the chromatin accessibility peaks that were present in both CytP and AgS cells showed a predominance of STAT and RUNX binding motifs in the opening peaks, almost in the same order as observed in CytP cells, and ATF family members in the closing peaks that occurred predominantly in CytP cells (Figure 5C; Supplementary Tables S5C and S6C). These findings indicate that chromatin accessibility to TF is markedly different in CytP and AgS cells while exhibiting a considerable degree of overlap.

### 2.6. Chromatin Accessibility of Genes Implicated in CD8^+^ T Cell Activation

**Figure 6:**
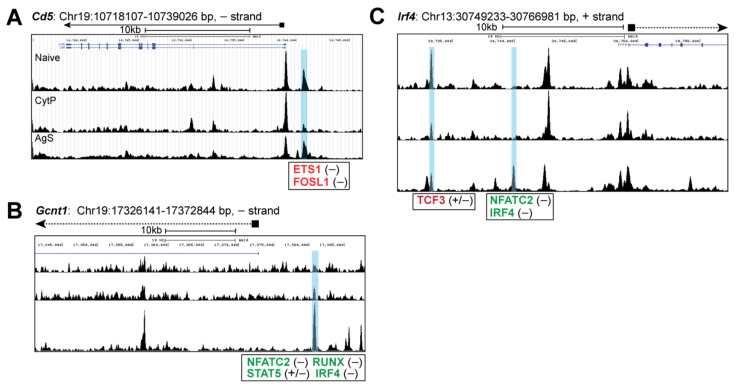
Chromatin accessibility of genes coding for proteins known to be modulated by cytokines in CD8^+^ T cells. Genome browser snapshots of chromatin accessibility signals near genes coding for proteins modulated by cytokine priming ((**A**), *Cd5*), Ag stimulation that promotes migration potential of memory CD8^+^ T cells ((**B**), *Gcnt1*), and the strength of pMHC-TCR interaction ((**C**), *Irf4*) in CytP and AgS cells.

Next, we examined chromatin accessibility near genes coding for proteins that are mechanistically linked to the increased Ag sensitivity of CytP cells, conferring migratory potential to memory CD8^+^ T cells or differentially modulated depending on the TCR affinity towards the antigenic peptide. We have previously shown that CD5, a negative regulator of TCR signaling is downmodulated in CytP cells [27,70]. Analysis of chromatin accessibility at the *Cd5* locus revealed a closing peak upstream of the *Cd5* gene that is predicted to harbor motifs for ETS1 (FDR = 1 × 10^−32^) and FosL1 (FDR = 1 × 10^−20^) in CytP cells that was not observed in AgS cells (Figure 6A). On the other hand, the locus containing *Gcnt1*, which is induced by IL-15 in memory CD8 T cells and codes for the enzyme that regulates 2-O-glycosylation of cell surface receptors to confer migratory potential [71], harbored an opening peak with binding motifs for NFATc, RUNX, IRF4 and STAT5 in AgS cells but not in CytP cells (Figure 6B). Finally, IRF4, a key TF implicated in modulating the expression of many genes in AgS cells in a quantitative manner in proportion to the strength of TCR stimulation [72], revealed an opening peak harboring an NFATc site in AgS cells but not in CytP cells, whereas a closing peak with the TCF3 binding motif occurred in both AgS and CytP cells (Figure 6C).

### 2.7. Expression of T Cell Activation Genes in CytP and AgS Cells

**Figure 7:**
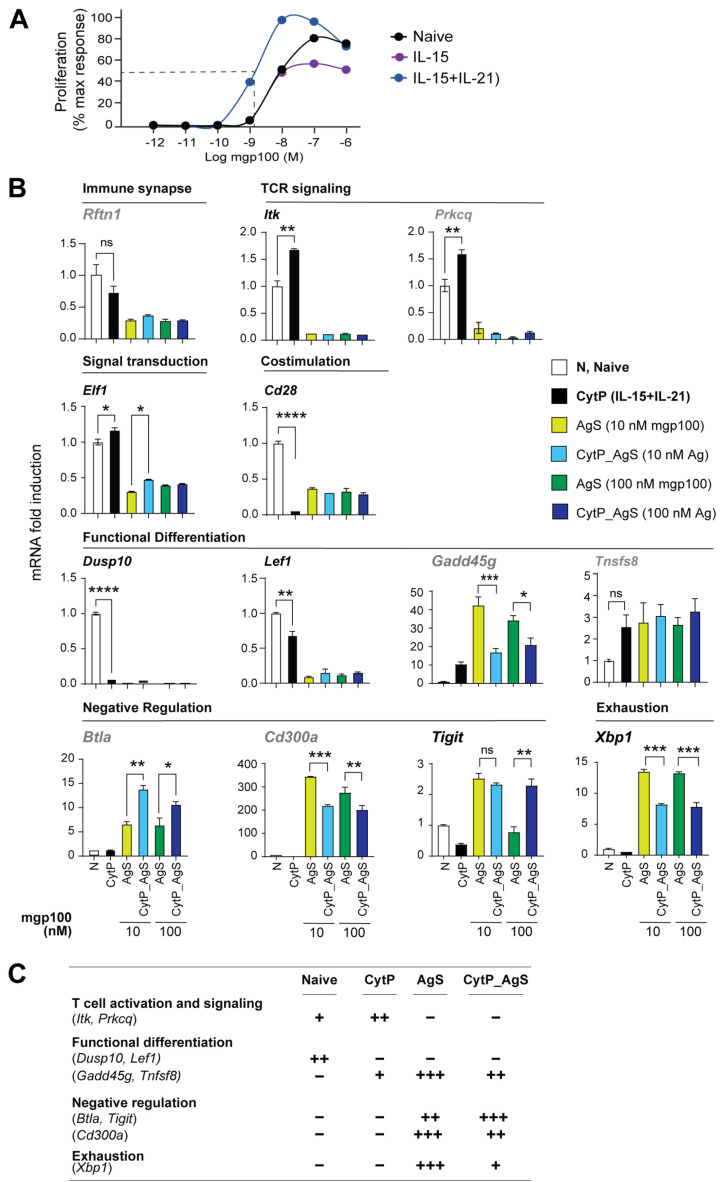
Expression of T cell activation genes in CytP and AgS cells. (**A**) Increased Ag sensitivity of naïve CD8^+^ T cells following cytokine priming. PMEL-1 naive CD8^+^ T cells were stimulated with IL-15 or IL-15+IL-21 for 48 h prior to stimulation with indicated concentrations of the cognate Ag peptide for 48 h. Cell proliferation was assessed by [3H]-thymidine incorporation and expressed % of maximal response. Representative data from one of two experiments are shown. (**B**) RT-qPCR analysis of the expression of select genes implicated in T cell activation, signaling, effector differentiation, negative regulation and exhaustion and identified in Table 2 in naïve (N), cytokine-primed (CytP) and Ag-stimulated (10 nM or 100 nM of PMEL-1 peptide) cells without (AgS) or with cytokine priming (CytP_AgS). Fold induction was calculated based on the expression level in naïve PML-1 cells. Mean + SEM for 3 independent experiments is shown. Comparison by one-way ANOVA with Tukey’s multiple comparison test. * *p* < 0.05, ** *p* < 0.01, *** *p* < 0.001, **** *p* < 0.0001. (**C**) A summary of gene expression patterns in naïve, cytokine-primed and Ag-stimulated cells.

We next determined how the limited changes in chromatin accessibility caused by cytokine priming correlated with gene expression by RT-qPCR. For this analysis, a third experimental group was included wherein CytP cells were stimulated with a suboptimal concentration of Ag (CytP_AgS). To determine the suboptimal Ag concentration at which cytokine priming caused a significant increase in cell proliferation, PMEL-1 cells primed with IL-15 alone or IL-15 and IL-21 were stimulated with mgp100_25-33_ peptide concentrations ranging from 10 mM to 1pM for 48 h (Figure 7A). A suboptimal peptide concentration of 10 nM and a 10-fold higher concentration (100 nM) were chosen to activate IL-15 + IL-21 stimulated cells for the evaluation of gene expression (Figure 7B and Supplementary Figure S5).

The *Rftn1* gene, which codes for a lipid raft resident protein, harbored two opening peaks in AgS cells and one opening peak in CytP cells (Table 2, Figure 4). *Rftn1* showed reduced expression in CytP cells and negligible expression in AgS cells (Figure 7). *Itk*, *Prkcq* and *Elf1* genes are implicated in T cell activation, cytokine gene expression and regulating the expression of TCR signaling components, respectively (Table 2). Among these, *Itk* and *Elf1* showed closing chromatin peaks whereas *Prkcq* showed opening chromatin peaks in both CytP and AgS cells (Table 2). However, cytokine priming alone caused a small but significant increase in the expression of all three genes that were completely repressed in AgS cells even with cytokine priming (Figure 7B). *Cd28,* coding for the costimulatory receptor, harbored closing peaks in CytP and AgS cells and was markedly suppressed in all stimulated cells. *Dusp10*, involved in the functional differentiation of T cells, harbored closed peaks in CytP and AgS cells and was similarly repressed in both populations (Table 2, Figure 7B). Genes that are implicated in maintaining the quiescence of naïve T cells namely *Foxp1* and *Lef1* harbored closing peaks in CytP and AgS cells, but only *Lef1* expression was significantly reduced in CytP cells and totally repressed in AgS cells (Table 2, Figure 7B and Supplementary Figure S5). *Gadd45g*, which promotes effector differentiation in CD8^+^ T cells and harbored opening peaks only in Ag cells, showed an appreciable increase in CytP cells and profound expression in AgS cells, but the latter was significantly reduced by prior cytokine priming (Table 2, Figure 7B). *Tnfsf8*, which promotes T cell proliferation and harbored opening peaks in CytP and AgS cells was discernibly induced in both groups, but this increase was not statistically significant. *Btla* and *Cd300a*, which are implicated in the negative regulation of T cell activation, showed opening peaks only in AgS cells (Table 2). However, both were markedly induced in AgS cells but showed opposite expression patterns in CytP_AgS cells, with a significant upregulation of *Btla* but downregulation of *Cd300a* (Figure 7B). Notable differences were found in the chromatin accessibility and expression of *Xbp1*, which is implicated in the exhaustion and induction of inhibitory receptors (Table 2). *Xbp1* was not expressed in naïve or CytP cells. However, *Xbp1* was highly induced in AgS cells despite harboring a closing peak, which was significantly attenuated by cytokine priming (Figure 7B).

A global view on the expression of candidate genes showing similar changes in chromatin accessibility peaks in CytP and AgS cells (Figure 7C) revealed that (i) cytokine priming induces changes in key genes involved in T cell activation that are suppressed following Ag stimulation, and (ii) AgS induces strong effector differentiation, whereas cytokine priming reduces the expression of the associated genes, and (iii) cytokine priming also upregulates certain negative regulatory genes more efficiently than Ag stimulation, and (iv) cytokine priming reduces the expression of genes involved in T cell exhaustion.

## 3. Discussion

Modulation of CD8^+^ T cell response by inflammatory cytokines produced by innate immune cells continues to be an area of intense scrutiny [11,26,73,74,75]. At least three types of cytokine-driven augmentation of CD8^+^ T cell responses have been recognized. First, the third signal cytokines type-I IFN (IFNαβ) and IL-12, which promote an efficient expansion and full activation of AgS CD8^+^ T cells, mediate these functions by sustaining the transcriptional program initiated by the TCR and costimulatory receptors via modulating histone acetylation at genetic loci that regulate cell survival, proliferation and effector functions [76]. The second cytokine-mediated modulation of CD8^+^ T cell functions is the IL-15-dependent increase in the Ag sensitivity of memory CD8^+^ T cells and their ability to migrate through tissues [25,71,77]. Whereas the latter function has been attributed to the increased expression of GCNT1, which promotes 2-O-glycosylation of cell surface molecules that interact with P- and E- selectins, the mechanistic basis for the IL-15-mediated increase in Ag sensitivity in memory cells remains to be elucidated. Third, the increase in Ag sensitivity of naïve CD8^+^ T cells mediated by inflammatory cytokines in synergy with homeostatic cytokines, which primes naïve T cells to respond to weak TCR agonists [19,27,78]. In this study, we show that cytokine priming modulates chromatin accessibility in a manner similar to Ag stimulation at several key genetic loci that harbor genes implicated in T cell activation, effector differentiation, negative regulation and exhaustion. However, CytP and AgS cells showed notable differences in the expression of these genes, with cytokine priming exerting a significant impact on AgS cells. Our findings support the notion that the priming of naïve CD8^+^ T cells by inflammatory cytokines not only confers a poised state for increased responsiveness to subsequent Ag encounter but also significantly modulates gene expression in antigen encountered cells that could enhance their functional fitness by reducing exhaustion without compromising regulatory controls.

Indirect evidence suggests an important role for inflammatory cytokines in modulating the Ag responsiveness of naïve CD8^+^ T cells. One of them is the heterogeneity in the magnitude of CD8^+^ T cell response towards cognate peptides. Following Ag stimulation in vivo, naïve OT-I cells vary in the expression of effector molecules, the propensity to differentiate into various effector subsets and the ability to kill of virus-infected cells [79,80,81]. As these studies used distinct viruses (influenza, vesicular stomatitis virus, murine cytomegalovirus) that expressed cognate OVA Ag, the heterogeneity of their responses could be at least partly explained by differential exposure to virus-induced cytokines and their impact on the strength of initial TCR activation. Such heterogeneity also occurs in the differentiation of CD4^+^ T cells with single TCR specificity in vivo, and was attributed to TCR signal strength as well as to environmental cues, particularly the cytokine milieu induced by adjuvants, although the effect of the latter can be nullified by quantitatively stronger TCR stimulation [82,83,84]. Supporting this argument, CD8^+^ T cells in human peripheral blood that are specific to cytomegalovirus, Epstein-Barr virus and influenza virus antigenic epitopes were shown to vary in the expression of CTL effector molecules, cytokine and chemokines [85]. As CD8^+^ T cells specific to the viral epitopes are unlikely to be monoclonal in origin, the TCR clonality could partially account for the variable responses. However, the cytokine context of their initial stimulation could also be a factor, which could account for the distinct response patterns observed against the Ag epitopes of the three different viruses. Therefore, there is a clear need to understand the molecular underpinnings that determine the role played by cytokines produced by innate immune responses in modulating the outcome of T cell activation [82,86].

A boost in the Ag-induced clonal expansion and effector CD8^+^ T cell differentiation by the third signal cytokines IFN-I and IL-12, and the IL-15-mediated increase in the functional avidity of memory CD8^+^ T cells were demonstrated using cytokine or cytokine receptor knockout mice [14,25,71,87]. On the other hand, evidence for the cytokine-induced increase in the functional avidity of the TCR in naïve CD8^+^ T cells mainly came from in vitro studies following stimulation with inflammatory cytokines in conjunction with IL-7 or IL-15 [19,23]. Obtaining genetic evidence for the cytokine priming of naïve CD8^+^ T cells was complicated by the redundancy of cytokine combinations that could cause this effect. Either IL-6 or IL-21 (and possibly other inflammatory cytokines) along with IL-7 or IL-15 could achieve the cytokine priming effect of boosting TCR functional avidity in vitro [19,23]. Such in vitro cytokine-primed autoreactive CD8^+^ T cells, stimulated with weak agonists of the TCR, were able to cause disease in a mouse model of autoimmune diabetes [23]. Moreover, PMEL-1 melanocyte Ag-specific TCR transgenic mice showed evidence of activation in vivo in the absence of SOCS1, the negative feedback regulator of IL-15 and IL-7 signaling, causing melanocyte destruction and inflammatory skin lesions [24]. These findings lend support to the notion that cytokines play a key role in virus-induced and lymphopenia-associated triggering of autoreactive CD8^+^ T cells [88,89,90].

Cytokine priming may play a role in activating CD8^+^ T cells bearing TCR with a low affinity toward pathogen-derived Ag and these responses are known to be important in pathogen elimination [91,92,93,94]. In fact, comparison of dodecamer and tetramer binding suggests that T cells expressing low-affinity TCRs are more abundant than those expressing high affinity TCR [95]. While the latter are retained in the secondary lymphoid organs through stable interactions with DC, the former are released into circulation as the concentrations of the pMHC complexes decrease over time and distance [96]. Although T cells bearing low-affinity TCR express effector molecules such as granzyme B and perforin and contribute to containing acute infections, they fail to expand and persist, in contrast to the high affinity clones [92]. However, during chronic infections, low affinity clones have been shown to predominate at later time points [97], and cytokine priming may play a role in these persistent immune responses.

Anti-tumor CD8^+^ T cells bearing low affinity TCR towards tumor associated Ag (TAA) and tumor-specific neo-Ag (TSA) may also benefit from cytokine priming. Due to the central and peripheral tolerance mechanisms, anti-tumor CD8^+^ T cells necessarily bear a low affinity TCR towards most TAAs [98]. Low affinity TCR clones are capable of containing a modest tumor burden [99,100] and increasing the TCR affinity by genetic manipulation results in off-target effects and autoimmunity [98]. We have shown that cytokine priming enables tumor Ag-specific CD8^+^ T cells to recognize and respond to an endogenous tumor Ag peptide [101]. Hence, cytokine priming could be used to expand antitumor CD8^+^ T cells bearing low affinity TCRs to circumvent the off-target toxicity associated with TCR engineering [102].

An essential step towards understanding and exploiting cytokine priming in protective immune responses against infections or cancers is to elucidate its mechanistic underpinnings. Even though our present study used one cytokine priming condition of IL-15 and IL-21 at a single timepoint on one TCR Tg CD8^+^ T cell model, our findings offer important insights into how CD8^+^ T cell priming by inflammatory cytokines could impact T cell responses following TCR signaling. Even though the transcriptional program activated by Ag as well as the TF that are involved have been extensively studied, chromatin accessibility assays are beginning to broaden our understanding of these changes at the genome level [103,104,105]. Consistent with these reports, the binding motifs for the bZIP containing TF were predominant in AgS cells. Notably, the binding motifs for two bZIP factors Jun-AP1 and Fosl2 were also enriched in CytP cells. Many bZIP factors co-operate with IRF4, which is induced by TCR stimulation and plays a fundamental role in CD8 T cell differentiation in proportion to the TCR affinity and signal strength [72,106]. The IRF4 binding motif figured among the top TF motifs near the opening peaks of AgS cells and was found near key genes that are known to be induced by TCR stimulation (Figure 3 and Figure 4). IL-15 was recently shown to enhance IRF4 expression in TCR-stimulated CD8 T cells [107]. We did not find a significant enrichment of the IRF4 binding motif in CytP cells (Supplementary Table S5A). However, the *Irf4* locus of CytP cells contained one significant change among the many induced by Ag stimulation, namely the loss of the binding peak for the transcriptional repressor TCF3 (E47A) (Figure 6) [108]. This suggests a poised state of the *Irf4* locus that could increase its induction in CytP cells following TCR stimulation, contributing to their increased Ag responsiveness.

Among the TF binding motifs shared by CytP cells with AgS cells, STAT and RUNX motifs figured predominantly near the common opening peaks (Figure 5C). The enrichment of the binding motifs for STAT5, STAT3 and STAT1, which are activated by the IL-2 family cytokines [109], in AgS cells could be explained by autocrine IL-2 signaling that occurs within 36 h of Ag stimulation. How the binding motifs for STAT4 and STAT6, which are activated during CD8^+^ T cell differentiation process induced by Ag stimulation [108], are enriched in CytP cells remains to be investigated. The Runt domain containing TF (RUNX) RUNX1, RUNX2 and RUNX3, which function as both transcriptional activators and repressors, are implicated in CD8^+^ T cell development and CTL differentiation [110,111,112,113]. The bZIP domain containing activating TF (ATF), which include several members including BATF, also function as both transcription activators and repressors and are implicated in the differentiation of activated CD8^+^ T cells [114,115]. Whereas RUNX binding motifs are shared between CytP and AgS cells near the opening peaks, the shared ATF motifs are found near the closing peaks (Figure 5). Clearly, the changes in chromatin accessibility of CytP cells have a vast potential to impact gene expression following Ag stimulation.

Even though chromatin accessibility peaks in general positively correlate with gene expression, deviations from this notion are frequently observed. Understanding such discordance between chromatin accessibility and gene expression is an area of intense scrutiny [116,117]. The cause of this discordance could be multifactorial, resulting from the activating and repressive nature of TF, cooperation with modifiers of TF activity, epigenetic states of the gene locus and chromatin dynamics that bring enhancers elements to the vicinity of TF accessible loci [118]. In this context, a recent study has shown that the high mobility group transcription factor TCF1, which binds to many genetic loci in naïve CD8^+^ T cells does not induce the expression of all genes in its vicinity but keeps them ‘ready’ for modulation [119]. Indeed, the induction of many of these genes during IL-15-driven homeostatic proliferation is associated with TCF1’s ability to interact with the transcriptional modifier CTCF, which is mobilized by cytokine stimulation [119]. This results in cooperative binding to DNA elements and changes in chromatin organization that could increase the accessibility to STAT5. We have documented increased STAT5 activation and its DNA binding in cytokine-primed cells [19]. Such higher order changes in chromatin dynamics could underlie the differential expression of genes in CytP and AgS cells despite an overlapping chromosome accessibility profile, and cytokine-induced modulation of their expression in AgS cells in synergistic or antagonistic manner (Table 2, Figure 7B). Even though the single-timepoint snapshot of chromatin accessibility and gene expression is a limitation of this study, our findings suggest that the cytokine priming of naïve CD8^+^ T cells could dynamically alter the binding and activity of TF that are modulated by Ag stimulation. Understanding these changes that regulate gene expression in the accessible chromatin of CytP cells will require a kinetic analysis of specific TF binding by advanced ChIPseq techniques at different timepoints of Ag stimulation in CytP cells.

## 4. Materials and Methods

### 4.1. Mice, Peptides and Cytokines

PMEL-1 TCR transgenic mice [120] were purchased from the Jackson Laboratory (Bar Harbor, ME, USA) and were used with the approval of the Université de Sherbrooke Ethics Committee for Animal Care and Use in accordance with guidelines established by the Canadian Council on Animal Care. The PMEL-1 melanoma antigen-derived peptide mgp100_25-33_ (EGSRNQDWL) [121] was custom synthesized by GenScript (Scotch Plains, NJ, USA) to more than 80% purity. Recombinant human IL-15 and mouse IL-21 were from R&D Systems (Minneapolis, MN, USA).

### 4.2. Cell Purification and Stimulation

Naïve CD8^+^ T cells were purified from the lymph node cells of PMEL-1 mice by negative selection using Invitrogen Magnisort CD8 Naïve T cell Enrichment kit (Thermo Fisher, Ottawa, ON, Canada, #8804-6825-74) following the manufacturer’s instructions. Cells were stimulated with IL-15 and IL-21 (both at 10 ng/mL) for 48 h or with the cognate antigenic peptide mgp100_25-33_ (1 μM) for 36 h in the presence of irradiated splenocytes from C57BL/6 mice as APC. CD8^+^ T cells from Ag-stimulated cultures were purified again by negative selection.

### 4.3. ATAC Sequencing

The ATACseq was performed following the methods described by Buenrostro et al., [122,123]. Unstimulated (naïve, N), cytokine stimulated (cytokine-primed, CytP) and Ag-stimulated (AgS) cells were washed in cold PBS, and 50,000 cells were suspended in lysis buffer (10 mM Tris-Cl, pH 7.4, 10 mM NaCl, 3 mM MgCl_2_ and 0.1% IGEPAL CA-630) at 4 °C for 5 min. The nuclei from two batches of cells for each condition were sedimented by centrifugation at 500 g for 10 min, pooled and DNA library was prepared using the Nextera DNA Library Prep kit (Illumina). Briefly, nuclei were resuspended in transposition reaction mix and incubated at 37 °C for 30 min. DNA fragments were purified using Qiagen Mini-Elute kit (Qiagen Cat# 28004). The DNA fragments were PCR amplified using custom Nextera primers containing different barcodes for naïve, CytP and AgS cells. The amplified fragment libraries were sequenced at the Université Laval (Quebec, QC, Canada) sequencing facility and analyzed at the Bioinformatics service platform of the Université de Sherbrooke.

Quality assessment of the amplified TN5 transposition fragment libraries showed an enrichment of single nucleosomes (200 bp peak) in naïve and CytP cells, whereas the AgS cells showed an enrichment of nucleosome dimers (400 bp peak) [122] (Supplementary Figure S6). Even though the latter may arise from annealing of the PCR products due to shortage of primers at the later PCR cycles that can be resolved by an additional round of PCR (https://dnatech.genomecenter.ucdavis.edu/faqs/ (accessed on 1 April 2020)), it contained libraries harboring sites for CCCTC-binding factor (CTCF), a highly conserved transcriptional regulator throughout the genome [124] again and sites encompassing transcriptional start sites (TSS) [122].

### 4.4. ATACseq Data Analysis

The raw ATACseq data were processed using Trimmomatic [125] before analysis using the ENCODE ATAC-seq pipeline for non-replicated data to obtain signal and peak files. Whereas naïve and CytP cells generated 34M and 30M paired-end (PE) reads, AgS cells generated 134M reads. From these data, 50,945, 53,297 and 135,836 peaks were identified in N, CytP and AgS cells, respectively. A peak atlas was generated using Bedops [126] to concatenate peak files and iteratively merge >75% overlapping peaks. The coverage of the peaks was determined using the coverage tool in BEDTools [127].

To identify opening and closing peaks in stimulated cells compared to naïve cells, the DESeq2 package, developed to allow for quantitative analysis based on strength rather than differential expression alone [128], was used. To avoid high fold change (FC) caused by a low count, the regularized log transformation (rlog) was used to define meaningful changes in chromatin accessibility. The distribution of rlogFC values for comparison between CytP versus N and AgS versus N cells are shown in Supplementary Figure S5A. Because the number of peaks detected in AgS cells were more than double compared to those of naïve or CytP cells, they were randomly assigned to two subgroups (AgS1, AgS2) of 85,640 and 86,748 peaks using seqtk (https://github.com/lh3/seqtk (accessed on 1 April 2020) in order to determine if there was any skewing of opening and closing peaks. These two subgroups behaved similarly to AgS cells in the principal component analysis (Figure 1B) and showed a similar enrichment of ATACseq reads around TSS and a similar pattern of opening and closing peaks (Supplementary Figure S5B,C). Hence, all peaks in AgS cells were used for subsequent analysis. The rlogFC threshold was set to log2(1.5) as log2(2) was too stringent to identify opening and closing peaks in CytP and AgS cells. The peaks were subsequently analyzed using HOMER [129] to identify genes that are nearest to the peaks and the transcription factor binding motifs. Gene positions obtained from the Mouse Genome informatics database (http://www.informatics.jax.org/ (accessed on 1 April 2020)) was used to interrogate the UCSC Mouse Genome browser mm10 assembly (http://ucscbrowser.genap.ca (accessed on 1 April 2020)) to visualize and capture snapshots of genome accessibility.

The STRING database [130] was used to study the interaction network analysis of proteins coded by genes in the opening and closing peaks and to identify gene ontology (GO) groups related to T cell activation, differentiation, effector functions and regulation. Only medium and high confidence interactions with a score above 0.40, supported by experiments, curated databases, protein homology, text mining and co-expression studies were considered for data interpretation.

### 4.5. Gene Expression and Statistical Analyses

Total RNA from purified CD8^+^ T cells (naïve or activated as indicated) was extracted using RNeasy Plus Mini Kit (Qiagen, Canada, Cat #74134), according to the manufacturer’s instructions. cDNA was synthetized from 200 μg of purified RNA using QuantiTect^®^ reverse transcription kit (Qiagen, Toronto, ON, Canada). Quantitative RT-PCR amplification reactions were carried out in CFX Connect real-time PCR detection system (Bio-Rad, Mississauga, ON, Canada) or QuantStudio 3 real-time PCR system (Thermo Fisher Scientific, Ottawa, ON, Canada) using SYBR Green Supermix (Bio-Rad, Mississauga, ON, Canada). The expression of indicated genes was measured using primers listed in Supplementary Table S7. Gene expression levels between samples were normalized based on the Cycle threshold (Ct) values compared to housekeeping gene m*36B4* (*Rplp0*) and the fold induction was calculated using the unstimulated cells as controls. Data were analyzed using the GraphPad Prism9 (San Diego, CA, USA). Statistical significance (*p* value) was calculated by one-way ANOVA with Tukey’s multiple comparison test. *p* values < 0.05 were considered significant.

## 5. Conclusions

In summary, even though the changes in chromatin accessibility induced by cytokine priming in naïve CD8^+^ T cells are 10-fold fewer than those induced by Ag stimulation, 30–50% of the changes induced in CytP cells also occur in AgS cells. Many of these changes occur near genes that are implicated in CD8^+^ T cell activation and differentiation and harbor consensus binding motifs for TF modulated by Ag stimulation. However, the similarities between CytP and AgS cells in chromatin accessibility is not reflected in gene expression. Whereas AgS caused profound changes in expression of most genes, CytP cells showed negligible changes. Nonetheless, cytokine priming modulated the expression of several genes in AgS cells, upregulating certain genes implicated in effector functions and downmodulating some genes that promote exhaustion. Our findings indicate that inflammatory cytokines induced during innate immune responses not only increase the Ag sensitivity of CD8^+^ T cells but also modulate gene expression to enhance their functional fitness and reduce exhaustion. This knowledge could be exploited for improving vaccination strategies and inducing antitumor immunity.

## Figures and Tables

**Table 1:** GO analysis of genes related to T lymphocyte activation and functions that show changes in chromosome accessibility peaks.

Term ID	Term Description	Observed Gene Count	Background Gene Count	FDR ^1^
(A) in cytokine-primed cells				
GO:0002376	immune system process	63	1703	2.19 × 10^−7^
GO:1902105	regulation of leukocyte differentiation	22	284	7.52 × 10^−7^
GO:0006955	immune response	35	914	0.00015
GO:0045582	positive regulation of T cell differentiation	9	90	0.00085
GO:0046634	regulation of alpha-beta T cell activation	9	98	0.0014
GO:0030098	lymphocyte differentiation	14	243	0.0016
GO:0045619	regulation of lymphocyte differentiation	11	171	0.0033
GO:0030217	T cell differentiation	10	154	0.0051
GO:2000516	positive regulation of CD4-positive, alpha-beta T cell activation	5	34	0.0059
GO:0046649	lymphocyte activation	16	378	0.009
GO:0046632	alpha-beta T cell differentiation	6	60	0.0091
GO:0042110	T cell activation	12	244	0.0118
GO:0050670	regulation of lymphocyte proliferation	11	215	0.014
GO:0070229	negative regulation of lymphocyte apoptotic process	4	39	0.0468
(B) in both cytokine-primed and Ag-stimulated cells				
GO:0051251	positive regulation of lymphocyte activation	18	253	2.70 × 10^−5^
GO:0050870	positive regulation of T cell activation	16	180	1.00 × 10^−5^
GO:0051249	regulation of lymphocyte activation	23	396	2.13 × 10^−5^
GO:0050863	regulation of T cell activation	20	287	1.05 × 10^−5^
GO:0045621	positive regulation of lymphocyte differentiation	10	103	0.00045
GO:0050852	T cell receptor signaling pathway	6	56	0.0069
GO:0046635	positive regulation of alpha-beta T cell activation	6	64	0.0118
GO:0050856	regulation of T cell receptor signaling pathway	5	39	0.0093
GO:0042129	regulation of T cell proliferation	9	162	0.0207

^1^ FDR, false discovery rate.

**Table 2:** Genes related to T lymphocyte activation, regulation and functions that are adjacent to the opening and closing ATACseq peaks in CytP and in both CytP and AgS CD8^+^ T cells.

Functional Category of Genes	ATACseq Peaks Modulated in:				
	CytP Cells	CytP & AgS Cells	Other Names	Functions in CD8^+^ or Other T Lymphocytes	Ref.
Immune synapse					
	*Rftn1* ^1^	*Rftn1* ^1^		Increases TCR signaling, needed for localisation of LCK to lipid rafts	[31]
Early TCR signaling					
	*Cd247* ^2^	*Cd247* ^2^	CD3z	Signaling component of the TCR:CD3 complex	[32]
	*Dpp4* ^1^	*Dpp4* ^1^	Dipeptidyl peptidase IV, CD26	Promotes T cell activation	[33]
	*Itk* ^2^	*Itk* ^2^	Tec family kinase	Required for efficient TCR signaling and T cell proliferation; Integrates TCR & CytR signaling	[34]
	*Prkcq* ^1^	*Prkcq* ^1^	PKC theta	Critical signal strength regulator for the activation of NF-kB, NF-AT, AP-1 transcription factors	[35]
Signal transduction to the nucleus					
	*Bcl10* ^2^	*Bcl10* ^2^	B-cell lymphoma/ leukemia 10	Essential for TCR-induced NF-kB activation	[36]
	*Chd7* ^1^	*Chd7* ^1^	Chromatin remodeler	T cell development in Zebrafish	[37]
	*Elf1* ^2^	*Elf1* ^2^		Regulates transcription of TCR ζ chain, LAT	[32,38]
	*Jun* ^1^			AP-1 family transcription factor, activated by TCR signaling	[39]
Cytoskeletal remodeling & cell migration					
	*Ccr1* ^1^			Promotes migration of activated T cells	[40,41]
	*Ccr7* ^1^	*Ccr7* ^1^		Controls CD8 T cell homeostasis by directing memory cells to IL-7-dependent niches	[42]
Co-stimulation					
	*Cd28* ^2^	*Cd28* ^2^		T cell co-stimulatory receptor	[43]
Functional differentiation					
	*Dusp10* ^2^	*Dusp10* ^2^	Mkp5	Facilitates proliferation & cytokine production	[44]
	*Foxp1* ^2^	*Foxp1* ^2^		Maintains quiescence in naive T cells	[45]
	*Gadd45g* ^1^			Promotes effector functions in CD8 T cells	[46]
	*Gpr18* ^2^	*Gpr18* ^2^		Controls effector-memory CD8 T cells	[47]
	*Il15ra*	*Il15ra* ^2^		Ligand-specificity subunit of IL-15R; controls memory CD8 T cell homeostasis	[48]
	*Il6st* ^2^	*Il6st* ^2^	gp130, IL-6 signal transducer	Needed for efficient generation of memory CD8 T cells	[49]
	*H2-Aa* ^2^		MHC-II antigen	Over-expressed in PD-1 KO CD8 T-CM phenotype cells	[50]
	*Lef1* ^2^	*Lef1* ^2^	Lymphoid enhancer binding factor 1; Possess HDAC activity	suppresses CD4 lineage genes in CD8 T cells; highly expressed in quiescent cells; downregulated by TCR and IL-15 signaling	[51]
	*Nfkbiz* ^1^	*Nfkbiz* ^1^	IkBz	promotes Th17 differentiation	[52]
	*Prkca* ^2^	*Prkca* ^2^	PKC alpha	Promotes Th17 resposne by upregulating IL-17A expression	[53]
	*Rara* ^2^	*Rara* ^2^		Essential for CD4 T cell activation and CD8 T cell survival	[54]
	*Stat5b* ^2^	*Stat5b* ^2^		Critical to maintain effector T cell responses	[55]
	*Tgfbr2* ^2^	*Tgfbr2* ^2^		Regulates IL-15-mediated CD8 T cell homeostasis	[56]
	*Tnfsf8* ^1^	*Tnfsf8* ^1^	TNF Superfamily Member 8; CD153; CD30L	Induces prolifertaion of T cells	[57]
Negative regulation					
	*Btla* ^1^		B and T-lymphocyte attenuator; CD272	Negative regulation of T cell activation	[58]
	*Cblb* ^2^	*Cblb* ^2^	Casitas B Lymphoma Proto-Oncogene B	Negative regulation of TCR signaling pathways	[59]
	*Cd101* ^2^			Negative regulation of T cell activation	[60]
	*Cd300a* ^1^			Negative regulator of T cell activation	[61]
	*Cd47* ^2^	*Cd47* ^2^		Promotes/inhibits T cell activation	[62]
	*Tigit* ^2^		CD115T	Suppresses CD8 T cell activation	[63]
Associated with Exhaustion					
	*Trib1* ^2^		Tribbles Pseudokinase 1	Expressed in hypofunctional CD8 T cells in tumors and during chronic infection	[64]
	*Xbp1* ^2^		X-box-binding protein-1	Effector T cell differentiation; Induces CD8 T cell exhaustion accompanied by increased expression of inhibitory receptors	[65,66]
Unknown functions in mature T cells					
	*Tmem64* ^2^	*Tmem64* ^2^		Positively regulates TNFSF11-mediated NF-AT-dependent Ca2+ signaling in osteoclasts; not yet implicated in Lymphocyte activation	[67]
	*Tnfsf11* ^2^		TNF Superfamily Member 11; RANKL; CD254	Suppresses effector cytokine production in group 3 innate lymphoid cells	[68]
	*Zfp609* ^1^	*Zfp609* ^1^		Represses *Rag* gene expression during T cell development	[69]

^1^ Gene names in grey are near opening ATACseq peaks. ^2^ Gene near closing peaks.

## Data Availability

All data generated or analyzed during this study are included in the Supplementary Information Files.

## Supplementary Materials

The following supporting information can be downloaded at: https://www.mdpi.com/article/10.3390/ijms232214122/s1.
