# Supplementary material for: Inflammatory Cytokines That Enhance Antigen Responsiveness of Naïve CD8+ T Lymphocytes Modulate Chromatin Accessibility of Genes Impacted by Antigen Stimulation
Source: Int J Mol Sci. 2022 Nov 16;23(22):14122. doi: 10.3390/ijms232214122 (PMC9698886; doi:10.3390/ijms232214122)
Supplement: Supplementary file 1 [file ijms-23-14122-s001.zip › Supplementary Figures_Edited.pdf]

Inflammatory cytokines that enhance antigen responsiveness of naïve CD8+ T lymphocytes modulate chromatin accessibility of genes impacted by antigen stimulation

Quenum et al.,

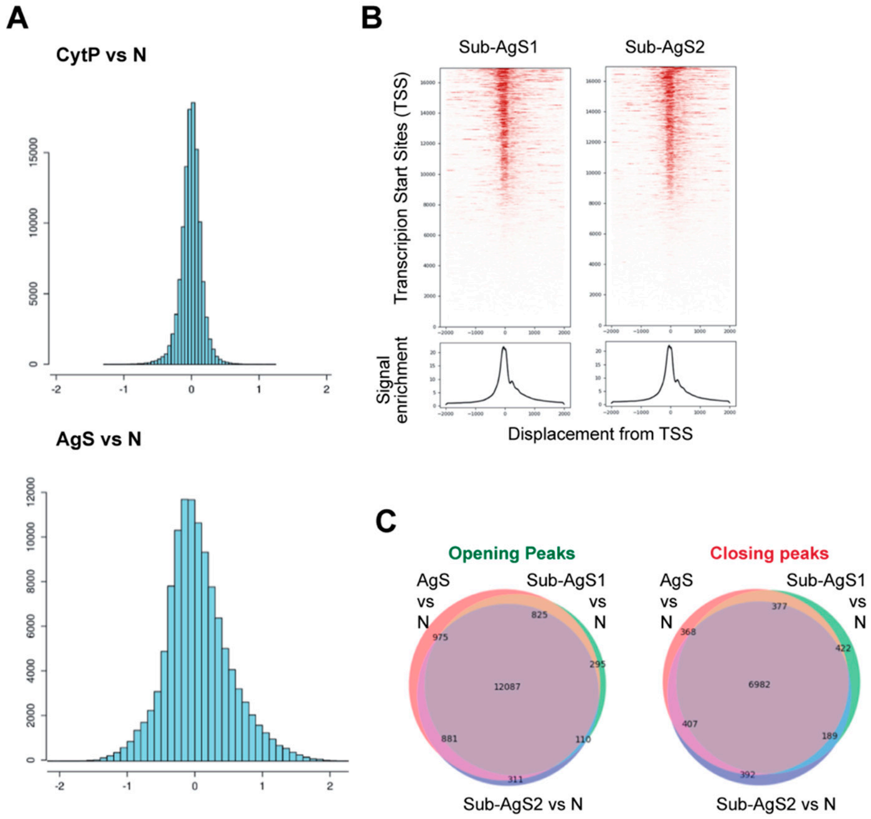

Supplementary Figure S1. Quality control of chromatin accessibility peaks in AgS cells. (A) Distribution of rlogFC values for comparison between CytP versus N and AgS versus N cells. (B) Fragment length distribution analysis of ATACseq reads from AgS cells randomly subgrouped to sub-AgS1 and sub-AgS2 reads. (C) Comparison of the opening and closing peaks in sub-AgS1 and sub-AgS2 ATACseq reads compared to naive cells.

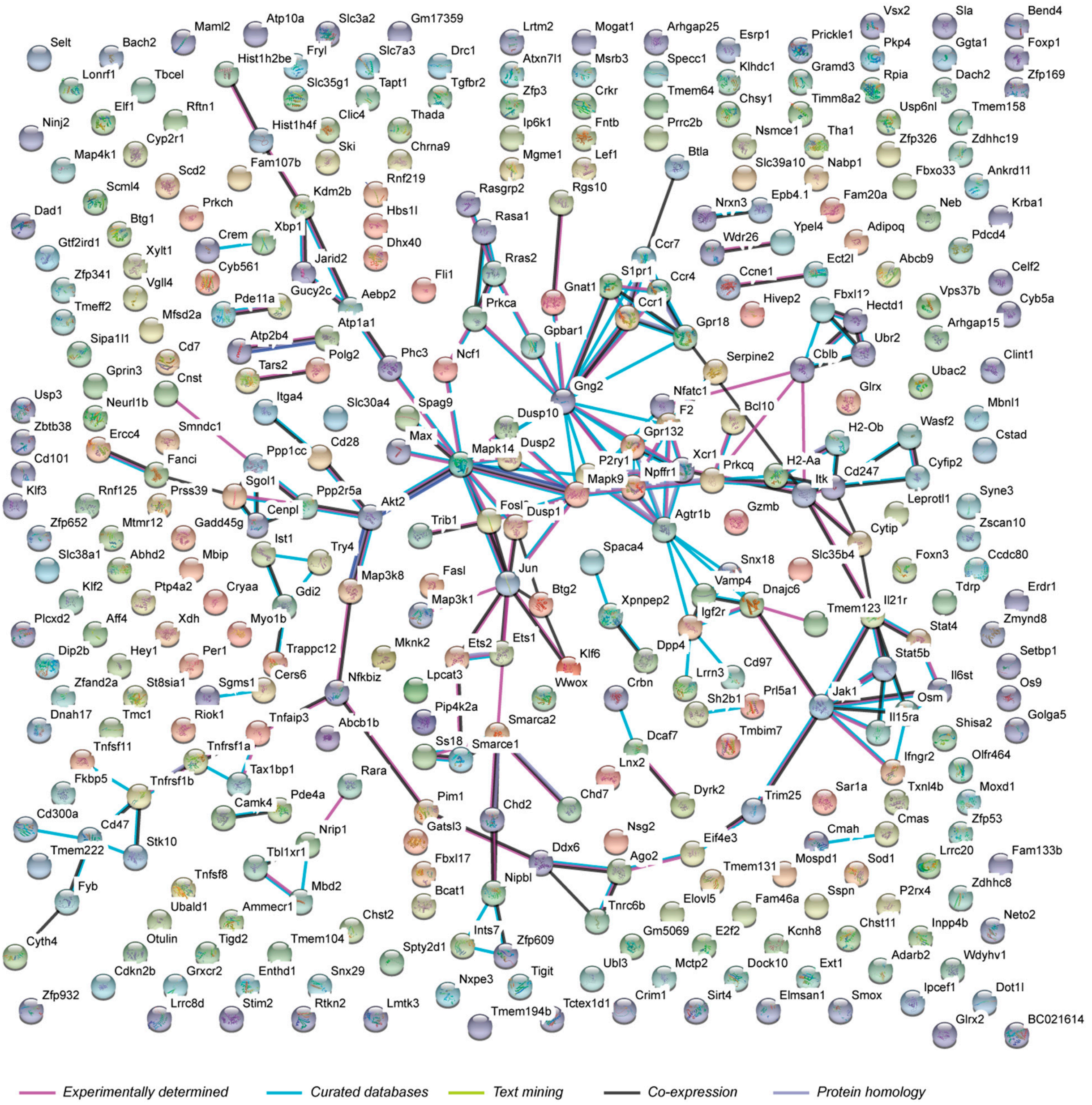

Supplementary Figure S2. Protein interaction network analysis of genes in the vicinity of the ATACseq peaks modulated in cytokine-primed PMEL-1 cells compared to naive cells.

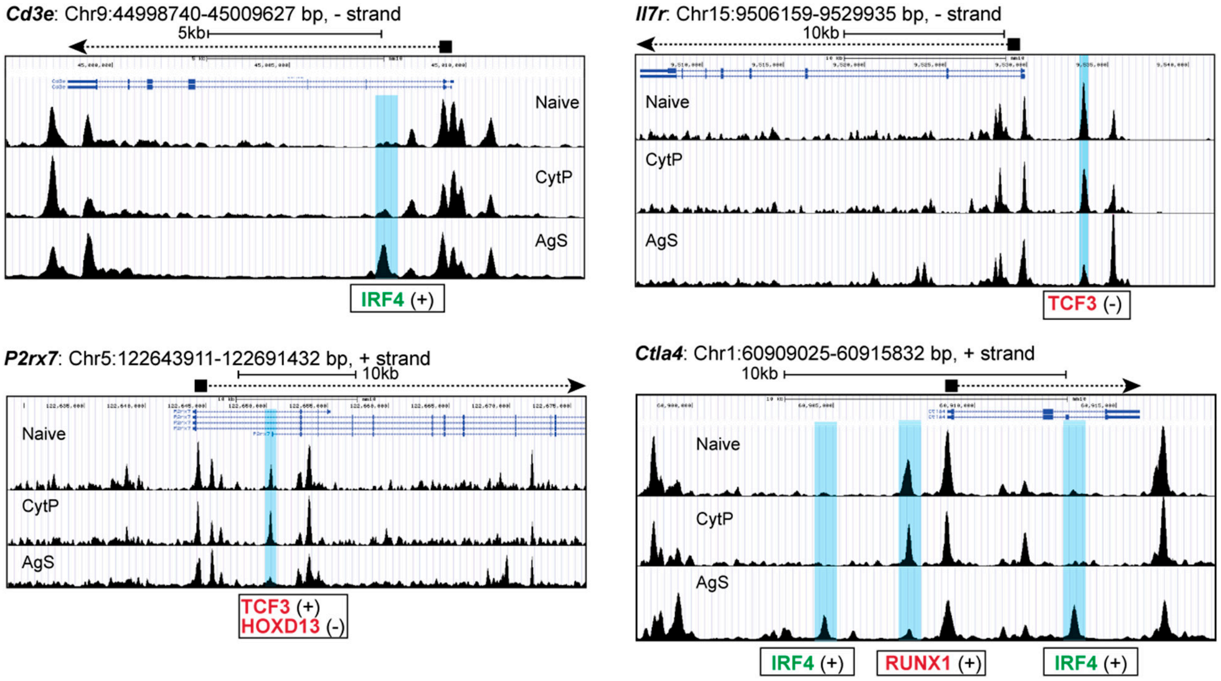

Supplementary Figure S3. Other examples of chromosome accessibility peaks opening (*Cd3e*), closing (*Il7ra, P2rx7*) or both opening and closing (*Ctla4*) in Ag-stimulated but not in cytokine-primed cells.

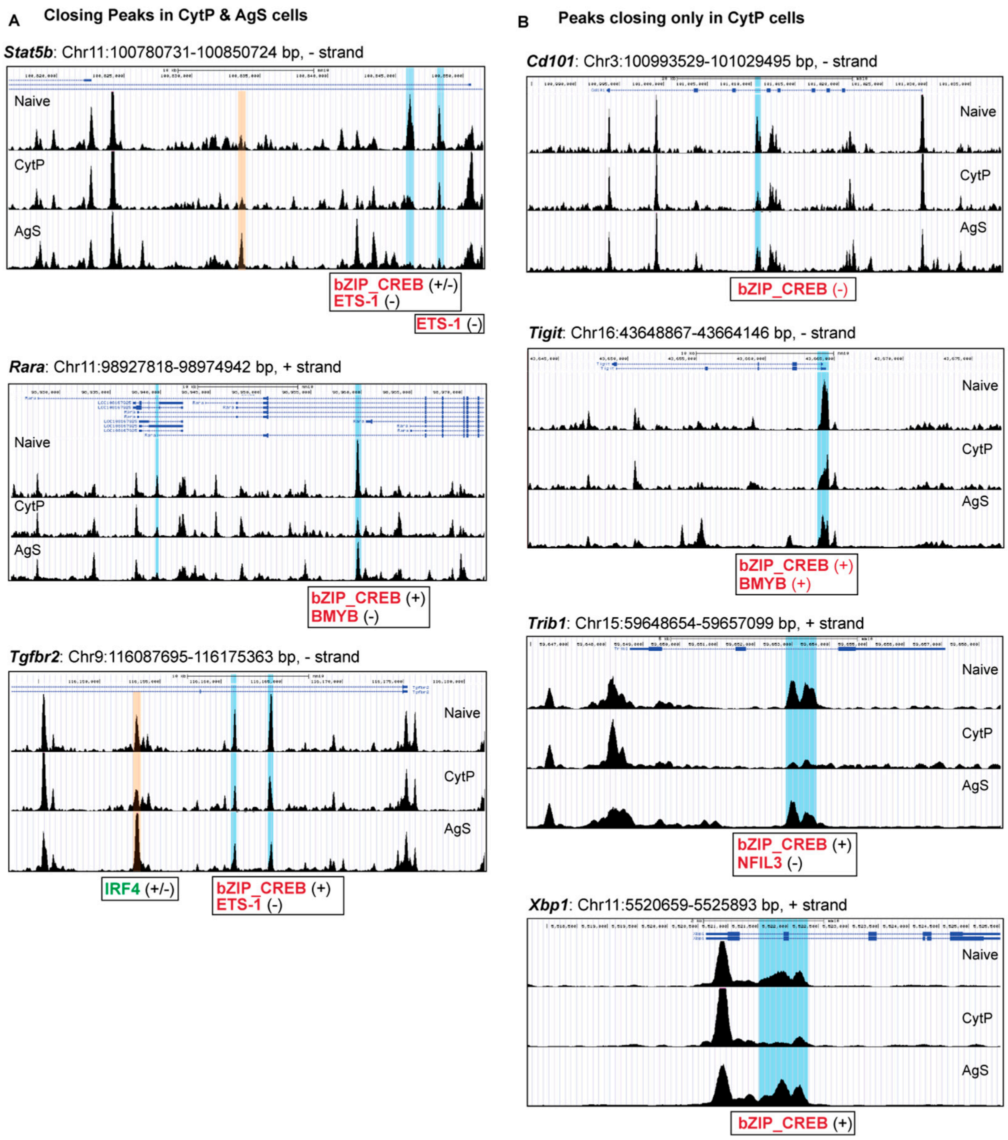

Supplementary Figure S4. Chromosome accessibility peaks that are closing in both CytP and in AgS cells (A) or only in AgS cells (B).

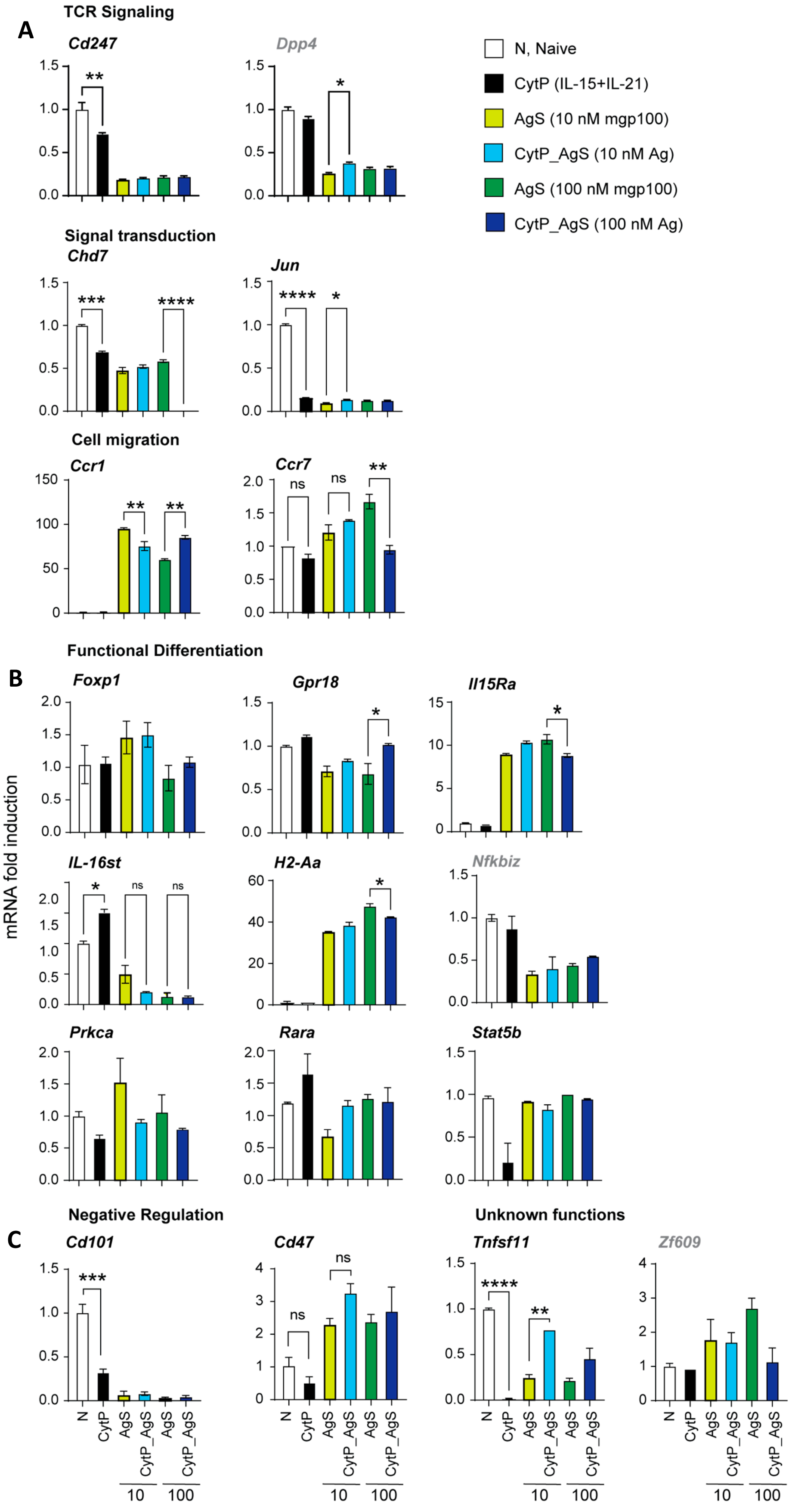
TCR Signaling
A
Cd247
Dpp4
N, Naive
CytP (IL-15+IL-21)
AgS (10 nM mgp100)
CytP_AgS (10 nM Ag)
AgS (100 nM mgp100)
CytP_AgS (100 nM Ag)
Signal transduction
Chd7
Jun
Cell migration
Ccr1
Ccr7
Functional Differentiation
B
Foxp1
Gpr18
Il15Ra
mRNA fold induction
IL-16st
H2-Aa
Nfkbiz
Prkca
Rara
Stat5b
Negative Regulation
Unknown functions
C
Cd101
Cd47
Tnfsf11
Zf609
N
CytP
AgS
CytP_AgS
AgS
CytP_AgS
10
100

Supplementary Figure S5. (A–C) RT-qPCR analysis of additional genes implicated in T cell activation, signaling, effector differentiation, negative regulation and exhaustion in naïve (N), cytokine-primed (CytP) and Ag-stimulated (10 or100 nM of PMEL-1 peptide) cells without (AgS) or with cytokine priming (CytP_AgS). Fold induction was calculated based on the expression level in naïve PML-1 cells. Mean + SEM for 3 independent experiments are shown. Comparison by one-way ANOVA with Tukey's multiple comparison test. *, *p* <0.05, ** <0.01, *** <0.001, **** <0.0001.

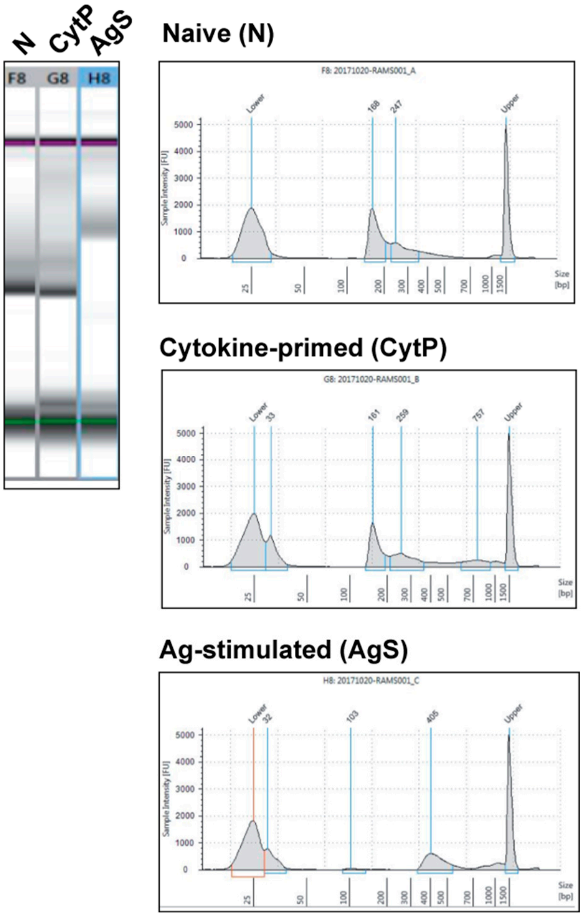

Supplementary Figure S6. Bioanalyzer quality assessment of ATACseq libraries of naive, cytokine-primed and Ag-stimulated PMEL-1 TCR transgenic CD8+ T lymphocytes.
